# Evaluation of material effects on three-dimensional cultured skeletal muscle cells for biohybrid robots

**DOI:** 10.3389/frobt.2026.1778864

**Published:** 2026-04-21

**Authors:** Hirono Ohashi, Shunsuke Shigaki, Seita Fujii, Masahiro Shimizu, Koh Hosoda

**Affiliations:** 1 Department of Agricultural Innovation for Sustainability, Tokyo University of Agriculture, Atsugi, Japan; 2 Graduate School of Engineering Science, Osaka University, Toyonaka, Japan; 3 Principles of Informatics Research Division, National Institute of Informatics, Chiyoda, Japan; 4 Department of Biodata Science, Nagahama Institute of Bio-Science and Technology, Nagahama, Japan; 5 Graduate School of Engineering, Kyoto University, Kyoto, Japan

**Keywords:** BiCAM, biohybrid robot, contractile force, extracellular matrix, responsiveness, three-dimensional cultured skeletal muscle cells

## Abstract

Robots are traditionally confined to controlled environments such as factories, where human interactions are limited. However, the demand for robots that are capable of collaborating with humans is increasing. To achieve symbiosis, integrating the physical flexibility and environmental adaptability of living organisms into robotic systems is crucial. An example of such a robot is a biohybrid robot driven by three-dimensional (3D) cultured skeletal muscle cells. These muscle cells, which are composed of myoblasts and an extracellular matrix (ECM), contract and generate force in response to external stimuli. The standardization of such 3D-cultured skeletal muscle cells is essential for practical applications. However, their complete standardization has not yet been achieved. The contractile force of 3D-cultured skeletal muscle cells produced via 3D printing is still insufficient for practical applications as actuators in biohybrid robots. In a previous study, we developed a simple fabrication method for 3D-cultured skeletal muscle cells. These bio-cultured artificial muscle (BiCAM) cells can control the shape and cell alignment of tissues. Differences in the composition of an ECM have been suggested to affect the contractile force of 3D skeletal muscle tissues; however, their impact on the response characteristics remains poorly understood. In this study, we investigated how the ECM composition influences the contractile force of 3D skeletal muscle cells in biohybrid robots as a step toward their eventual standardization. Compared with tissues cultured under MF conditions, in which electrically induced contraction was previously confirmed, tissues cultured under CM conditions exhibited an approximately two-fold greater contractile force at voltage amplitudes of 10 and 30 V. Furthermore, the fabrication success rate was 100 % under CM conditions but only 62.5–70 % under other ECM conditions. In contrast, although CM tissues generated larger forces, tissues cultured under MgF and CMg conditions exhibited higher-frequency response. These findings demonstrated that the BiCAM is a viable actuator and offers new possibilities for the design of biohybrid robots.

## 1 Introduction

As computers, actuators, and sensors hsvr become more sophisticated, the performance of robots has improved dramatically, and robots play essential roles, particularly in line work in factories. In known environments, such as factories, traditional rigid robots can operate at high speed and with high precision, supporting the work of humans. However, when they encounter unknown environments or situations that they have not yet learned, they are unable to demonstrate sufficient performance. Generally, living organisms cannot perform repetitive tasks as quickly and precisely as robots; however living organisms are highly adaptable and can respond flexibly to unknown environments. This is generally defined as adaptability, and attempts to have been made to incorporate the adaptability of living organisms into robotic systems. One of the most prominent trends in this field is the development of soft robots (biohybrid robots) powered by myocytes (Webster-Wood et al., 2017; Yuan et al., 2023; Garmroudi et al., 2025).

Cells, which are the building blocks of living organisms, are known to change their morphology, lineage, and other properties depending on their living environment (Engler et al., 2006). By leveraging this environmental adaptability and using myocytes, which are the building blocks of living organisms, robots can adapt to their environment more effectively. For example, 3D-cultured skeletal muscle cells are a type of power source for biohybrid robots and have the advantages of low heat loss and high efficiency in terms of energy consumption (Kovač, 2014). Methods using electrical stimulation (Cvetkovic et al., 2014) and light (Raman et al., 2016) have been proposed to externally control 3D-cultured skeletal muscle cells. In addition, to use them in more practical environments, biohybrid robots that can be driven in air (Morimoto et al., 2020), remotely controlled biohybrid robots (Kim et al., 2023), and biohybrid hands (Ren et al., 2025) have been developed.

Various biohybrid robots driven by 3D-cultured skeletal muscle cells have been developed (Morimoto et al., 2018; Kabumoto et al., 2013; Guix et al., 2021). Standardizing 3D-cultured skeletal muscle cells is essential for practical applications; however, complete standardization has not yet been achieved (Raman et al., 2017). Furthermore, while 3D-cultured skeletal muscle cells have been developed using 3D printing, which promises to produce a variety of shapes, they have not yet been used in biohybrid robots for manipulation because of their lack of contractile force and their medical developmental purpose (Mestre et al., 2019; Alave Reyes-Furrer et al., 2021). Establishing a simple and reproducible method for fabricating 3D-cultured skeletal muscle cells of various shapes would likely improve the diversity and performance of the actuators.

In our previous study, we developed an easy fabrication method for 3D-cultured skeletal muscle cells with high design flexibility, including the use of a 3D bioprinter, and called the resulting 3D-cultured skeletal muscle cells bio-cultured artificial muscle (BiCAM) (Ohashi et al., 2023). These 3D-cultured skeletal muscle cells were fabricated using molds and dishes with pins, which contributed to the shape and cell alignment. Previous studies have demonstrated that the gelation process determines the tissue morphology, with the gel edges pinned to guide cell alignment for efficient force generation, whereas tissues without pins do not exhibit such alignment (Ohashi et al., 2023). In addition to these 3D muscle tissue fabrication techniques, it is essential to consider the extracellular matrix (ECM) composition as a factor that influences actuator performance.

3D-cultured skeletal muscle cells are generally fabricated by mixing myoblasts with an ECM and culturing them in three dimensions. Because the ECM plays an important role in maintaining the tissue structure and intercellular connections, the contractile force of 3D skeletal muscle cells is generally recognized to vary with the composition of the ECM (Chitcholtan et al., 2013; Shima et al., 2018; Soares et al., 2012). For 3D-skeletal muscle cells to function effectively as actuators in biohybrid robots, they must generate a sufficient level of contractile force while being capable of appropriately tracking and responding to periodic external stimuli. Differences in ECM composition have been suggested to affect the contractile force of 3D-cultured skeletal muscle tissues. However, how the ECM composition influences their response characteristics remains unclear, and quantitative evaluation is still lacking.

Here, we investigated how the ECM composition influences the contractile performance of 3D-cultured skeletal muscle cells used as biohybrid robot actuators as a step toward their eventual standardization. If these relationships can be quantitatively elucidated, it would be possible to embed BiCAMs with suitably tailored response properties into different regions of a biohybrid robot. For example, high-force-generating constructs can be deployed where a large actuation is required, whereas others optimized for periodic motion can be used where rhythmic activity is essential, thereby substantially enhancing the overall locomotor performance of a robot.

## 2 Materials and methods

### 2.1 Preparation of molds and Petri dishes

The molds and culture dishes were fabricated according to previously reported protocols (Ohashi et al., 2023). In the present study, only I-shaped 3D cultured skeletal muscle constructs (BiCAMs) were prepared. To culture the I-shaped BiCAMs, culture dishes were designed such that the pins were positioned at both ends along the longitudinal axis. First, a base for pin insertion was fabricated from ABS resin using a 3D printer (Guider2, Flashforge 3D Technology Co., Ltd., Zhejiang, China). The fabricated base was placed in a 10-cm culture dish, into which two shafts serving as pins (diameter: 0.8 mm; length: 8–10 mm) were inserted. Polydimethylsiloxane (PDMS) was then poured into the dish and cured by heating at 50 ° C for 6 h. The distance between the two pins was set as 20 mm.

In addition, the molds were fabricated by laser cutting polyoxymethylene (POM) sheets using a laser cutter (FABOOL Laser CO2, SmartDIYs, Japan), and their design was created using an Autodesk Inventor (Autodesk Inc., CA, USA). The groove of the fabricated mold was a rectangle measuring 25 mm in length and 3.5 mm in width, which were identical to the dimensions of the mold used for the I-shaped BiCAM reported in our previous study (Ohashi et al., 2023).

### 2.2 Myoblast

The primary cultured mouse myoblasts (MYB02) used for the fabrication of BiCAMs were purchased from Cosmo Bio Co., Ltd. (Tokyo, Japan).

### 2.3 Fabrication of BiCAM

The ECM compositions of the 3D cultured skeletal muscle cells fabricated in this study are summarized in Table 1. The collagen, Matrigel, and fibrinogen used in this study were selected because they have already been used as ECMs in 3D cultured skeletal muscle cells for biohybrid robots (Cvetkovic et al., 2014; Morimoto et al., 2018). The procedure for fabricating the BiCAMs with different ECM compositions is described below. The fundamental protocol was based on a previously reported method (Ohashi et al., 2023). We note that ECMs are mixed together, the final concentration of each component is inevitably diluted.

- C

**TABLE 1:** Solution compositions in each condition.

Solution compositions (v/v, (%))							
ECM condition		ECM					Growth medium
		Collagen	Matrigel GFR	Matrigel	Fibrinogen	Thrombin	
i)	C	100	-	-	-	-	-
ii)	MgF	-	60	-	20	4	16
iii)	CMg	45	45	-	-	-	10
iv)	CM	45	-	45	-	-	10

Cryopreserved myoblast-containing tubes were retrieved from −80° C storage and thawed. After thawing, the culture medium was added and the cells were centrifuged to remove the supernatant. The cell pellet ( $3.0\times 10^{6}$ cells) was then resuspended in 1,000 μ L of collagen solution prepared using a collagen gel culture kit (A. Cellmatrix Type I-A; B. 10 × MEM; C. Reconstitution Buffer; Nitta Gelatin Inc., Japan), yielding a homogeneous cell–gel mixture. Subsequently, 200 μ L of the cell–ECM mixture was dispensed into molds mounted in pin-equipped culture dishes. The constructs were incubated at 37 ° C under 5 % ${\text{CO}}_{2}$ for 40 min to allow gelation, after which the molds were removed.

Growth medium consisting of high-glucose DMEM supplemented with 10 % fetal bovine serum (FBS) and 1 % penicillin-streptomycin was supplemented with ACA (A2504-25G, Sigma-Aldrich, MO, USA) at a final concentration of 1 % (v/v), corresponding to 150 mg ACA per 1 mL of medium. A total of 10 mL of this ACA-containing growth medium was added, and the constructs were incubated at 37 ° C and 5 % ${\text{CO}}_{2}$ . The culture medium was replaced on days 3–4, and on day 6, the medium was replaced with the differentiation medium. The differentiation medium consisted of MYBDM (Cosmo Bio Co., Ltd., Japan) supplemented with an insulin solution (1 μ L per 1 mL of low-glucose DMEM (D6046-500ML, Sigma-Aldrich) containing 1 mg insulin and ACA added to a final concentration of 1 % (v/v), corresponding to 200 mg ACA per 1 mL of low-glucose DMEM.

ii. MgF

Cryopreserved myoblasts were thawed as described above, centrifuged after the addition of the medium, and the supernatant was discarded. Matrigel (Corning 356231), which is a fibrinogen solution prepared by dissolving 20 mg of fibrinogen (Sigma-Aldrich F3879) in 1 mL of low-glucose DMEM, and a thrombin solution containing 50 units of thrombin (Sigma-Aldrich T7009) in 1 mL of 0.1 % BSA were prepared in advance. To the cell suspension ( $3.0\times 10^{6}$ cells), 164 μ L of medium, 180 μ L of Matrigel, and 40 μ L of the thrombin solution were added, followed by the addition of 420 μ L of Matrigel and 200 μ L of the fibrinogen solution. The mixture was gently homogenized to obtain a uniform cell–ECM mixture. Subsequent culture and differentiation procedures were performed in the same manner as previously described.

iii. CMg

Collagen (450 μ L), which was prepared using a collagen gel culture kit (A. Cellmatrix Type I-A; B. 10 × MEM; C. Reconstitution Buffer; Nitta Gelatin Inc., Japan), was added to the cell suspension ( $3.0\times 10^{6}$ cells) and thoroughly mixed. Subsequently, 100 μ L of culture medium and 450 μ L of Matrigel (Corning 356231) were added and gently mixed to obtain a homogeneous cell-ECM mixture. Culture and differentiation procedures were performed as described above.

iv. CM

The basic fabrication procedure was identical to that used for CMg. Collagen (450 μ L), which was prepared using the collagen gel culture kit (A. Cellmatrix Type I-A; B. 10 × MEM; C. Reconstitution Buffer; Nitta Gelatin Inc., Japan), was added to the cell suspension ( $3.0\times 10^{6}$ cells) and thoroughly mixed. Subsequently, 100 μ L of culture medium and 450 μ L of Matrigel (Corning 356237) were added and mixed to yield a homogeneous cell–ECM mixture. Subsequent culture and differentiation steps were performed as previously described.

### 2.4 BiCAM contractile force measurement

Contractile force measurements were conducted 8 days after fabrication of the tissues. This experiment was conducted according to the method described by Ikeda et al. The contractile force of each BiCAM sample was quantified using a microforce sensor (AE801, Kronex, CA, USA). Because the sensor operated by detecting changes in electrical resistance induced by strain, calibration was performed by establishing a relationship between the voltage output and contractile force (mN). A shaft identical in size to the pins used for the BiCAM fixation was mounted on the tip of the sensor. Specifically, weights of 50, 100, 200, and 500 mg were gently placed on the shaft using tweezers, and the corresponding voltage signals during loading were recorded. The acquired voltage data were processed in MATLAB (MathWorks, MA, USA); after detrending to remove baseline drift, a Butterworth low-pass filter (cutoff frequency: 10 Hz) was applied to eliminate high-frequency noise. For each weight, the mean voltage difference over a 0.25 s interval before and after loading was calculated twice, and these values were used to derive the calibration equation relating the voltage change to the applied force.

One of the pins fixed the BiCAM was removed, and the shaft attached to the sensor was inserted into the resulting hole. The 3D-cultured skeletal muscle cells were immersed in high-glucose DMEM (D6046-500ML, Sigma-Aldrich, USA), and electrical stimulation was applied via platinum electrodes positioned at both ends with an interelectrode distance of 75 mm. Electrical stimulation was controlled using a microcontroller (Arduino Uno, Arduino SRL, Italy). The sensor output voltage and stimulation timing signals were routed to an analog-to-digital converter and sampled at 1 kHz. The acquired voltage data were processed in MATLAB by detrending to remove baseline drift, followed by the application of a low-pass Butterworth filter (cutoff frequency: 10 Hz) to suppress high-frequency noise. Subsequently, the difference between the voltage peak during contraction and pre-stimulus baseline was calculated and converted into a force value using the calibration equation. However, if the value was negative, the contractile force was set to zero.

The stimulation parameters were defined as follows: (1) duration: 10–500 ms at 10 V and 1 Hz; (2) voltage: 2–30 V at 100 ms and 1 Hz; and (3) frequency: 1–8 Hz at 100 ms and 10 V. The contractile force responses under each condition were quantitatively evaluated.

### 2.5 Tissue analysis by antibody staining

Immunofluorescence staining was performed on day 8 after fabrication of the BiCAMs. To facilitate efficient staining, each actuator was sectioned into four to six pieces, rinsed twice with PBS, and immersed in PBS for 5 min, which was repeated twice. The sections were fixed in PBS containing 2 % formaldehyde for 60 min at 4° C. After rinsing twices with PBS, the samples were immersed twice in PBS for 5 min. Sections were then permeabilized by incubation in PBS containing 0.5 % Triton X-100 (160-24751, FUJIFILM Wako Pure Chemical Corp., Japan) for 5 min. For antigen retrieval, the samples were transferred to 10 mM sodium citrate buffer (pH 6.0) containing 0.05 % Triton X-100 and incubated at 95° C for 30 min. Subsequently, blocking was performed overnight at 4° C in PBS supplemented with bovine serum albumin (BSA; 015-27053, FUJIFILM Wako Pure Chemical Corp., Japan) and 0.3 % Triton X-100.

The following day, the samples were rinsed twice with PBS and immersed twice in PBS for 5 min each. The sections were then incubated with an anti-myosin heavy chain antibody (1:300; MAB4470, R & D Systems) diluted in PBST at 37° C for 60 min. After two PBS rinses followed by two 5-min PBS immersions, the samples were incubated overnight at 4° C in the dark with a secondary antibody, donkey anti-mouse IgG (1:1000; A32787, Invitrogen, MA, USA), diluted in PBST.

Following two additional PBS rinses and two 5-min PBS immersions, the sections were mounted in PBS within perforated silicone rubber holders and imaged using a confocal laser scanning microscope (FV1200-IX83, Olympus, Japan).

### 2.6 Data analysis

All of the statistical analyses were performed using SPSS (version 26, IBM Japan, Ltd., Japan). For each condition, 15–20 data points were used to assess normality. When normality was not satisfied, the Kruskal–Wallis test was applied, followed by pairwise comparisons using the Dunn–Bonferroni *post hoc* procedure.

Box plots for each data point were drawn using R software (version 4.4.1; R Core Team). The system identification toolbox of MATLAB (2023b, MathWorks, MA, USA) was used to model the BiCAM, and Bode plots were created using MATLAB.

## 3 Results

### 3.1 Force measurement of BiCAMs under each ECM conditions

**FIGURE 1:**
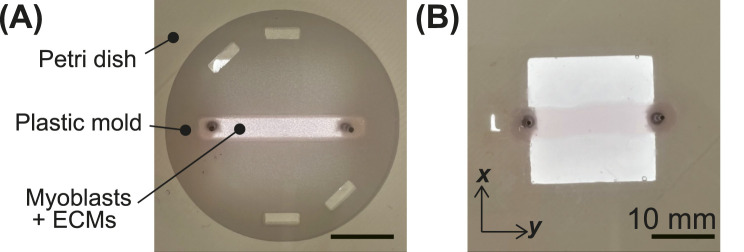
Overview of a bio-cultured artificial muscle (BiCAM). **(A)** BiCAM before mold removal. **(B)** BiCAM after mold removal.

To examine the suitability of BiCAMs as actuators for biohybrid robots, we modified the ECM composition during the fabrication and measured the contractile force under electrical stimulation. The four ECM compositions are listed in Table 1. Conditions i) C and ii) MgF corresponded to those employed in a previous study (Ohashi et al., 2023). Condition iii) consisted of collagen and growth factor-reduced Matrigel (GFR Matrigel; CMg condition). It was selected based on prior evidence that Matrigel promotes myoblast fusion, which is essential for myofiber development (Grefte et al., 2012; Cheng et al., 2014), whereas collagen constitutes the principal extracellular matrix component of biological skeletal muscles (Csapo et al., 2020). Under condition iv) comprising collagen and Matrigel (CM condition), non-growth factor-reduced Matrigel was used instead. Because this formulation contained a higher abundance of growth factors than the GFR Matrigel (Corning Inc, 2023), we hypothesized that these factors might contribute to the enhanced contractile force. The CMg and CM conditions were not optimized in this study. Using these four ECM conditions, I-shaped BiCAMs were fabricated, as illustrated in Figure 1, and their contractile forces were systematically evaluated. During the force measurements, the stimulation duration, voltage, and frequency were varied, and the resulting changes in the contractile force were comprehensively analyzed.

**FIGURE 2:**
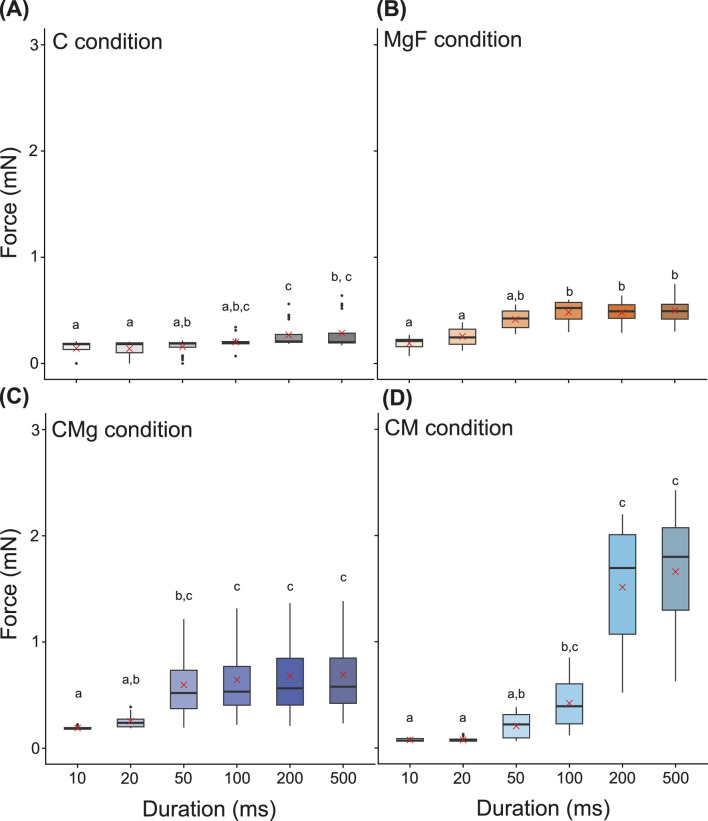
Box plot of contraction force in each duration time. **(A–D)** Duration time = 10, 20, 50, 100, 200, 500 ms, Voltage amplitude = 10 V, Stimulus pulse frequency = 1 Hz. The top of Boxes indicate the third quartile. The bottom of Boxes indicate the first quartile. The line in Boxes indicate the median. The upper whisker and the lower whisker indicate upper fence and lower fence, respectively. Dots show outliers. Red cross mark indicates mean value. Statistical analysis was performed for each ECM condition. Post-hoc pairwise comparisons were conducted using Dunn’s method with Bonferroni adjustment, following a significant Kruskal–Wallis test. Different letters above the bars indicate significant differences ( p< 0.05); identical letters indicate no significant difference. n= 20 (five replicates per sample). BiCAMs cultured under different ECM conditions: **(A)** C, **(B)** MgF, **(C)** CMg, and **(D)** CM.

First, to identify the appropriate stimulation duration, the contractile forces were measured under pulse durations of 10, 20, 50, 100, 200, and 500 ms at 10 V and 1 Hz (Figure 2). Under the C condition, no clear contractile response was observed in one of the four samples when stimulation durations of 10–20 ms were applied (see also the Supplementary Material). In contrast, contractions were observed at 10 ms under all other ECM conditions. In all cases, longer stimulation durations resulted in greater contractile forces: approximately 0.1–0.3 mN for the i) C condition, 0.2–0.5 mN for the ii) MgF condition, 0.2–0.7 mN for the iii) CMg condition, and 0.1–1.7 mN for the iv) CM condition. Notably, a significant increase in force was observed at stimulation durations of 100 ms or longer. However, when the duration was extended to 500 ms, contractions were also observed after the stimulus offset (data not shown). Based on these observations, a stimulation duration of 100–200 ms was deemed appropriate.

**FIGURE 3:**
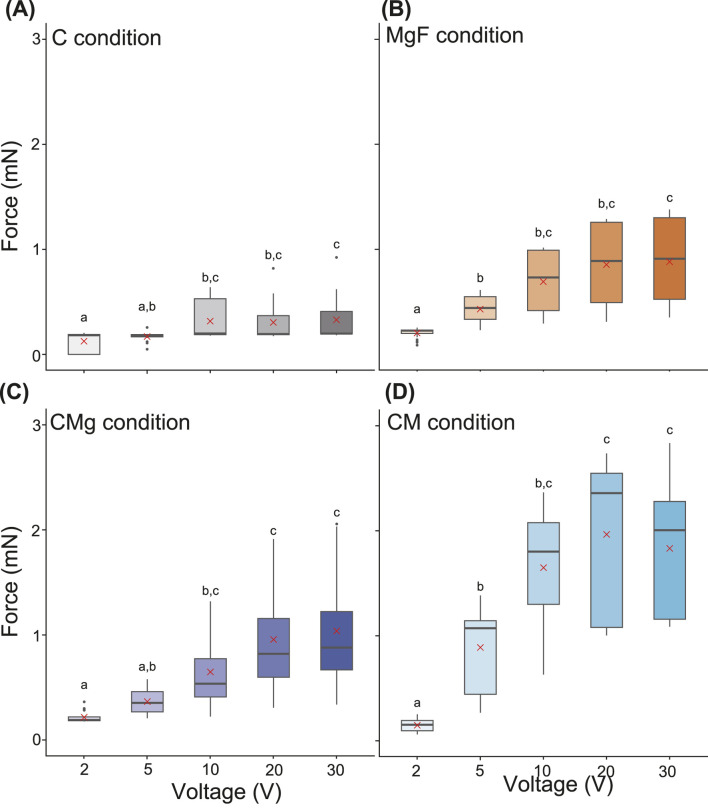
Box plot of contraction force in each voltage amplitude. **(A–D)** Duration time = 100 ms, Voltage amplitude = 2, 5, 10, 20, 30 V, Stimulus pulse frequency = 1 Hz. The top of Boxes indicate the third quartile. The bottom of Boxes indicate the first quartile. The line in Boxes indicate the median. The upper whisker and the lower whisker indicate upper fence and lower fence, respectively. Dots show outliers. Red cross mark indicates mean value. Statistical analysis was performed for each ECM condition. Post-hoc pairwise comparisons were conducted using Dunn’s method with Bonferroni adjustment, following a significant Kruskal–Wallis test. Different letters above the bars indicate significant differences ( p< 0.05); identical letters indicate no significant difference. **(A)** n= 15, **(B–D)** n= 20 (five replicates per sample). BiCAMs cultured under **(A)** C, **(B)** MgF, **(C)** CMg, and **(D)** CM conditions.

Next, the effect of the stimulation voltage on the contractile force was examined while keeping the pulse duration constant at 100 ms (Figure 3). Under all the ECM conditions, higher voltages resulted in larger contractile forces, although the response saturated at approximately 30 V. In contrast, repetitive or multiple contractions were not observed at higher voltages (Supplementary Movie 1). These findings indicated that increasing the voltage is an effective means of eliciting stronger contractions in BiCAMs. When comparing the mean contractile forces across the ECM conditions, the values ranged from 0.1 to 0.3 mN for the i) C condition, 0.2–0.9 mN for the ii) MgF condition, 0.2–1.0 mN for the iii) CMg condition, and 0.1–2.3 mN (Max: 3.5 mN) for the iv) CM condition.

**FIGURE 4:**
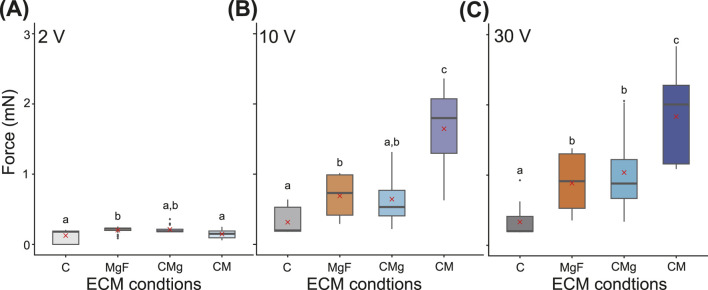
Box plot of comparison of contraction forces under ECM conditions. **(A–C)** The top of Boxes indicate the third quartile. The bottom of Boxes indicate the first quartile. The line in Boxes indicate the median. The upper whisker and the lower whisker indicate upper fence and lower fence, respectively. Dots show outliers. Red cross mark indicates mean value. Statistical analysis was performed for each voltage condition. Post-hoc pairwise comparisons were conducted using Dunn’s method with Bonferroni adjustment, following a significant Kruskal–Wallis test. Different letters above the bars indicate significant differences ( p< 0.05); identical letters indicate no significant difference. Voltage amplitude were **(A)** 2 V, **(B)** 10 V, and **(C)** 30 V. i) C condition: n= 15, ii) MgF, iii) CMg, and iv) CM conditions: n= 20 (five replicates per sample).

Finally, the contractile forces were compared across ECM conditions at fixed voltages (Figure 4). At 2 V, all of the ECM conditions yielded similar forces approximately 0.2 mN, with the ii) MgF condition exhibiting the highest value (Figure 4A). In contrast, at 10 and 30 V, a different trend emerged, with the iv) CM condition producing the greatest contractile force. Specifically, at 30 V, the mean contractile forces were 0.25 mN for the i) C condition, 0.77 mN for the ii) MgF condition, 1.12 mN for the iii) CMg condition, and 2.5 mN for the iv) CM condition.

Furthermore fabrication success rate was 100 % under CM conditions, but was 62.5–70 % under other ECM conditions (Supplementary File 2). These results suggest that the contractile force of the BiCAM can be optimized by electrical stimulation and that the CM condition is suitable for both the fabrication and functional performance of the construct (Figures 2–4).

### 3.2 Evaluation of response characteristics through modeling of BiCAMs

We found that the magnitude of the force generated differed depending on the ECM composition. When BiCAMs are embedded in biohybrid robots, periodic electrical stimulation can be applied to induce a continuous motion. In such scenarios, the rapidity with which the actuator responds to electrical stimuli is a critical determinant of the performance. Accordingly, from a control-theoretic perspective, we investigated how the responsiveness varied as a function of the ECM composition.

**FIGURE 5:**
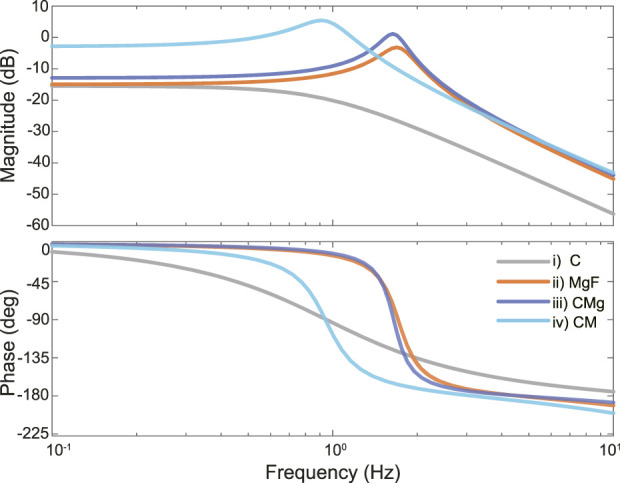
Frequency response characteristics of modeled BiCAM for each ECM condition. Bode plots were created based on representative samples for each ECM condition. i) Collagen (C) conditions are indicated by gray dotted lines, ii) Matrigel GFR + fibrinogen (MgF) conditions by orange lines, iii) Collagen + Matrigel GFR (CMg) conditions by dark blue dashed lines, and iv) Collagen + Matrigel (CM) conditions by a combination of light blue dotted and dashed lines, respectively.

BiCAMs fabricated with different ECMs were subjected to electrical stimulation at frequencies of 1, 2.5, 5, and 8 Hz, and their time-series responses were recorded. The time-domain data were subsequently modeled using a state-space representation. Because previous studies have demonstrated that muscle dynamics can be approximated by a mass-spring-damper system (Ohashi et al., 2023; Winters, 1990), the present model adopted a second-order state-space formulation. System identification was performed using the System Identification Toolbox in MATLAB (2023b; MathWorks, Inc., MA, USA). The resulting models for each ECM condition were used to analyze the frequency–response characteristics, as shown in Figure 5, where the upper panels depict the gain plots, and the lower panels show the phase plots.

An inspection of the gain plots indicates that the bandwidths of the respective BiCAMs were approximately i) 0.8 Hz, ii) 2.6 Hz, iii) 2.5 Hz, and iv) 1.4 Hz. The bandwidth magnitude served as a quantitative indicator of responsiveness. In this context, responsiveness denotes the extent to which the output can rapidly track and respond to variations in the input signal, and the BiCAMs reflected the ability to promptly generate the intended force in response to periodic electrical stimulation.

Among the tested conditions, the iv) CM condition produced the largest force under low-frequency or single-pulse electrical stimulation. In contrast, under a periodic input, the ii) MgF and iii) CMg conditions exhibited superior performances. Notably, a comparison of the peak frequencies indicates that the iii) CMg condition was capable of generating a larger output force than the ii) MgF condition, suggesting that, overall, BiCAMs fabricated with the CMg ECM composition are the most suitable choice.

Taken together, these results demonstrate that simply altering the ECM composition enables substantial modulation of not only the force magnitude, but also the frequency response characteristics. This finding implies that by appropriately selecting the ECM, it is possible to endow biohybrid robots with the desired dynamical or locomotive properties.

### 3.3 Histological analysis of BiCAMs under each ECM condition

**FIGURE 6:**
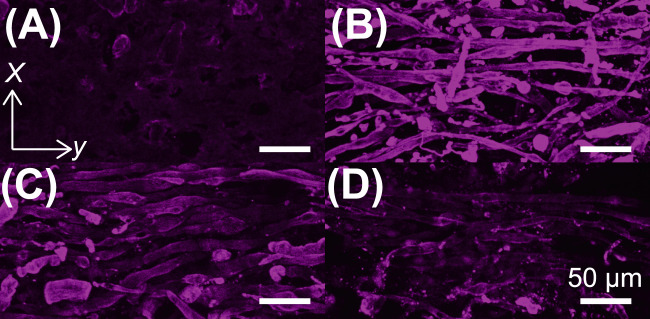
Confocal image of a skeletal muscle tissue immunostained with myosin heavy chains (MHC). **(A–D)** Magenta indicates myosin heavy chains. Scale bar, 50 μ m. **(A)** BiCAMs cultured with **(A)** C condition, **(B)** MgF condition, **(C)** CMg condition, **(D)** CM condition.

Finally, we performed a histological analysis using antibody staining to explore the factors underlying the differences in the force magnitudes and frequency responses (Figure 6). Based on the results of previous studies (Ohashi et al., 2023), the muscle cells in the I-shaped BiCAM were expected to be aligned along the longitudinal axis (y-axis). As a result, the i) C condition, which had low contractile force, was not observed to have muscle cell fibers, and the muscle tissue development was insufficient. In contrast, under the other conditions, muscle cell orientation was observed along the y-axis. However, no significant differences were observed in the width or degree of orientation of the muscle cells, and the reasons for the different tissue characteristics could not be identified. It has been reported that MHC expression patterns vary with muscle development in myoblast cells (Brown et al., 2012). Therefore, to further explore the differences in these properties, a gene-expression comparison is needed.

## 4 Discussion

In this study, we analyzed the contractile force of a BiCAM, which was created using a simple 3D-cultured skeletal muscle cell production method developed in previous research, with the goal of applying it to a biohybrid robot (Figures 2–5). Specifically, we measured the contractile force while varying the ECM conditions to determine the ECM conditions that maximized the contractile force (Figures 2–4). To achieve periodic movements, we investigated the response characteristics of each BiCAM under intermittent electrical stimulation (Figure 5). In this study, we analyzed the function of the BiCAM under four ECM conditions: i) collagen only (C), ii) Matrigel with GFR and fibrinogen with GFR (MgF), iii) collagen and Matrigel with GFR (CMg), and iv) collagen and Matrigel (CM).

When we focused on the contractile forces of the BiCAMs cultured with different ECMs, we found that the i) C condition had a low contractile force, the ii) MgF and iii) CMg conditions had intermediate contractile forces, and the iv) CM condition had the highest contractile force (Figure 3). These results suggest that the difference in contractile force was due to the growth factors (GFs) contained within the Matrigel and the combination of Matrigel and collagen. According to Corning’s product information, Matrigel contains IGF (average Matrigel: 15.6 ng/mL, typical GFR Matrigel: 5 ng/mL) and PDGF (average Matrigel: 12 pg/mL, typical GFR Matrigel: < 5 pg/mL) compared to GFR Matrigel. IGF and PDGF-B have been reported to be involved in muscle development (Yoshida and Delafontaine, 2020; Hamaguchi et al., 2023). In the future, analyses of these convergent pathways may reveal the molecular basis of the contractile properties observed in these results.

Next, we discuss responsiveness, which is an index of the response speed to an electrical stimulation input. Under the iv) CM condition, which produced the strongest contractile force, responsiveness was not necessarily the best. In contrast, the i) MgF and iii) CMg conditions, which produced moderate contractile forces, had better responsiveness values. In other words, the ECM affected not only the contractile force but also the responsiveness (Figure 5), suggesting that by selecting an appropriate ECM, it would be possible to design a BiCAM that can freely adjust the force and responsiveness. The skeletal muscles of vertebrates comprise, two types of muscle fibers: type 1 and type 2. Type 1 (slow-twitch) fibers have a slow contraction speed and low contractile force, but are endurant (Plotkin et al., 2021). On the other hand, type 2 (fast-twitch) fibers have a faster contraction speed and greater contractile force than slow-twitch muscles but lack endurance (Smerdu et al., 1994; Schiaffino and Reggiani, 2011). These fibers are not uniformly distributed, and their composition varies in each muscle; importantly, organisms can change the composition of muscle fibers to allow muscles to adapt to different uses (Talbot and Maves, 2016). This implies that living organisms change the muscle fibers that make up their muscles depending on how they are used. The same applies to 3D-cultured skeletal muscle cells for biohybrid robots, and it is important to provide muscles with the appropriate properties for each robot part. By varying the ECM composition, we quantitatively characterized the differences in the BiCAM response properties, thereby providing valuable insights for biohybrid robot design. These results suggest that ECM-dependent changes in response characteristics may reflect differences in muscle-fiber type. However, because no significant differences were detected in the muscle-cell width or alignment, future work will employ gene expression analysis and/or immunostaining to estimate the fiber-type composition. Furthermore, a limitation of this study was that the response characteristics of the BiCAMs were only evaluated under four ECM conditions. BiCAMs with a wide variety of characteristics can be designed by varying the ECM composition. In future, we will conduct a more detailed analysis and develop a biohybrid robot that can appropriately position BiCAMs according to their functions and purposes.

The contractile forces of the 3D-cultured skeletal muscle cells used in biohybrid robots typically range from several hundred micronewtons to approximately 10 mN (Cvetkovic et al., 2014; Morimoto et al., 2018; Guix et al., 2021). The BiCAM exhibited a contractile force of approximately 1–2 mN under various stimulation and ECM conditions (Figures 2–4). Based on this, we conclude that the BiCAM has a contractile force comparable to that of the muscles used in conventional biohybrid robots and can be used as an actuator in biohybrid robots. Methods have been developed to achieve a greater contractile force by increasing the number of tissue layers (Morimoto et al., 2018) and assembling multiple muscle bundles into a roll (Ren et al., 2025). Combining these methods will likely enable significant improvements in the contractile force of BiCAMs in the future. Although these methods may improve the contractile force, longitudinal evaluation is essential because biohybrid robots require long-term operation. In the present study, the functional analysis was performed only on day 8. Future studies should assess the post-stimulation lifespan and durability of the fabricated tissues.

Previous studies have primarily focused on simplified methods for fabricating 3D-cultured skeletal muscle cells, and the relationship between the ECM composition and their response characteristics has not been fully discussed. By integrating our shape-controllable fabrication method (Ohashi et al., 2023) with ECM-based tuning of the response properties, BiCAMs with the desired performances can be produced independently of the shape. The primary aims in this study had been to clarify relationships between the ECM composition and functional and response properties, we also found that ECM influences not only the functional and response properties but also the culture process itself. This is likely attributable to ECM-dependent changes in the physical properties of the tissues. For example, the fabrication success rate was lower under the ii) MgF and iii) CMg conditions, where the time constant was shorter (Figure 5). This may reflect stronger elastic elements that generate larger restoring forces, occasionally causing BiCAM to tear during culture. These results suggest that the physical properties of BiCAM can potentially be tuned by varying the ECM composition, which may represent an important factor for future standardization of BiCAM. Nevertheless, because living cells are used, inherent variability among cells is unavoidable. Therefore, it will also be important to establish techniques that maintain consistent reproducibility of contractile force regardless of the maturation state of the cells (see Supplementary Material). Systematic investigation from these perspectives will be an important direction for future research. In conclusion, the BiCAMs produced using our method offer new possibilities for biohybrid robot design and may enable the stable fabrication of 3D-cultured skeletal muscle cells.

## Data Availability

The original contributions presented in the study are included in the article/Supplementary Material, further inquiries can be directed to the corresponding author.
